# A One Health real-time surveillance system for nowcasting *Campylobacter* gastrointestinal illness outbreaks, Norway, week 30 2010 to week 11 2022

**DOI:** 10.2807/1560-7917.ES.2022.27.43.2101121

**Published:** 2022-10-27

**Authors:** David Swanson, Clemence Koren, Petter Hopp, Malin E Jonsson, Gunnar Isaksson Rø, Richard A White, Gry Marysol Grøneng

**Affiliations:** 1 Norwegian Institute of Public Health, Oslo, Norway; 2 Department of Biostatistics, University of Oslo, Oslo, Norway; 3 Norwegian Veterinary Institute, Ås, Norway

**Keywords:** campylobacter, gastrointestinal illness, outbreak nowcasting, website, One Health

## Abstract

**Background:**

*Campylobacter* is a leading cause of food and waterborne illness. Monitoring and modelling *Campylobacter* at chicken broiler farms, combined with weather pattern surveillance, can aid nowcasting of human gastrointestinal (GI) illness outbreaks. Near real-time sharing of data and model results with health authorities can help increase potential outbreak responsiveness.

**Aims:**

To leverage data on weather and *Campylobacter* on broiler farms to build a risk model for possible human *Campylobacter* outbreaks and to communicate risk assessments with health authorities.

**Methods:**

We developed a spatio-temporal random effects model for weekly GI illness consultations in Norwegian municipalities with *Campylobacter* monitoring and weather data from week 30 2010 to 11 2022 to give 1-week nowcasts of GI illness outbreaks. The approach combined a municipality random effects baseline model for seasonally-adjusted GI illness with a second model for peak deviations from that baseline. Model results are communicated to national and local stakeholders through an interactive website: Sykdomspulsen One Health.

**Results:**

Lagged temperature and precipitation covariates, as well as 2-week-lagged positive *Campylobacter* sampling in broilers, were associated with higher levels of GI consultations. Significant inter-municipality variability in outbreak nowcasts were observed.

**Conclusions:**

*Campylobacter* surveillance in broilers can be useful in GI illness outbreak nowcasting. Surveillance of *Campylobacter* along potential pathways from the environment to illness such as via water system monitoring may improve nowcasting. A One Health system that communicates near real-time surveillance data and nowcast changes in risk to health professionals facilitates the prevention of *Campylobacter* outbreaks and reduces impact on human health.

## Introduction

In syndromic and data-driven infectious disease surveillance, health indicators are used to facilitate early detection of outbreaks [1]. Syndromic surveillance is based on non-laboratory confirmed information, and so using several data sources and models for outbreak detection is often desired since both the sensitivity and specificity of one data source can be suboptimal [2,3]. A One Health perspective is increasingly acknowledged as important for surveillance and preparedness, given that approximately 75% of emerging pathogens affecting humans are regarded as zoonotic [4]. Thus, combining available data from animal and human health with environmental sectors in a risk model is an important aspect of improved surveillance.

One well-established source of food- and waterborne gastrointestinal (GI) illness in humans is *Campylobacter* spp [5,6]. In Norway, there have been over 35,000 samples from humans positive for *Campylobacter* since 2004 [7], including at least six GI illness outbreaks with between 3 and 2000 cases, where *Campylobacter* was confirmed as the source of infection [8]. In the EU, there are over 246,000 human *Campylobacter* cases reported annually [9]. Most human cases of Campylobacteriosis are sporadic with an unknown source and can occur through consumption of contaminated food or water, contact with animals, or from a contaminated environment. With respect to food contamination, farmed chicken broilers are prone to *Campylobacter* infection [10-12]. There are still knowledge gaps in the transmission routes of *Campylobacter* to broilers [13], but it is suggested that the outdoor environment is a major source of *Campylobacter* in broiler flocks.

Unmonitored *Campylobacter* in the environment, especially water systems, is a concern for human infection. The influence of temperature on *Campylobacter* abundance in both broilers and humans is known [14,15], and subsequent leakage of the pathogen into water systems because of precipitation events is a hypothesis for GI illness outbreaks as well as transport of the bacterium into broiler houses [16,17]. Using the number and proportion of *Campylobacter*-positive broiler flocks as a proxy of increased *Campylobacter* in the environment, combined with weather data, could provide an opportunity to foresee and prevent future outbreaks. 

Real time visualisation of surveillance data and stakeholders’ ability to access these data are crucial for the utility of early disease detection and limiting the impact of an outbreak. Interactive websites for this purpose are increasingly used in several countries. Examples are the FoodNet Fast, Foodborne Diseases Active Surveillance Network, at the Centers for Disease Control and Prevention (CDC) (https://www.cdc.gov/foodnet/foodnet-fast.html) and the European surveillance portal EpiPulse [18,19]. In Norway, a closed interactive website (Sykdomspulsen for kommunehelsetjenesten) for municipality doctors and county leaders featuring surveillance of coronavirus disease (COVID-19) and respiratory and GI illness has been ongoing since 2020 [20,21]. By providing timely outbreak nowcasts at the municipal level to central health authorities in Norway through an interactive website, one can improve municipalities’ public health responses.

The aims of this study were to improve the surveillance of GI illnesses by building statistical nowcasting models using weather data and broiler farm *Campylobacter* surveillance data, and disseminate these nowcasts via a website where warnings are generated when appropriate so that action can be taken when needed.

## Methods

### Study design

The study was designed as a spatial-temporal time series analysis with week and municipality as the units. The outcome was weekly counts of GI consultations within all Norwegian municipalities (n = 356) from week 30 2010 (starting 26 July) to week 11 2022 (starting 14 March) and modelled using lagged values of *Campylobacter* detection percentages and different weather pattern features within each municipality. Data sources and their extent are shown in Table 1 with each described in detail below.

**Table 1:** Data sources used in the study of real-time surveillance system for nowcasting increased gastrointestinal illness consultations, Norway, week 30 2010–week 11 2022 (n = 356 municipalities)

Data source	One Health component	Data owner	Frequency	Geographical area
Gastrointestinal illness consultation data	Human (aged 30–64 years)	Norwegian Health Directorate	Daily	All municipalities in Norway
*Campylobacter* testing in broiler flocks	Animal	Norwegian Food Safety Authority	Weekly	All municipalities in Norway where at least one broiler farm is located (n = 107)
Precipitation	Environment	Meteorological Institute	Daily	All municipalities in Norway
Temperature	Environment	Meteorological Institute	Daily	All municipalities in Norway

### Data sources

#### Consultation data for human gastrointestinal illness

Consultation data consisted of all physician consultations at clinics and urgent care facilities among all individuals aged 30 to 64 years in Norway from week 30 2010 to week 11 2022 (Table 1). This age group was chosen because of (i) the existing granularity of age strata in the data and (ii) a concern that younger and older age strata would be more enriched for non-*Campylobacter* infections such as norovirus, adenovirus and sapovirus. During a consultation, one or more diagnosis code(s) based on the International Classification of Primary Care (ICPC-2) [22] system are assigned to every patient contact and are electronically submitted to the Norwegian Directorate of Health to receive reimbursement for consultations [23]. The data are electronically sent to the Norwegian Institute of Public Health (NIPH) on a continuous basis. NIPH is responsible for the surveillance of food and waterborne illness in humans in Norway. When data are received, they are automatically cleaned and aggregated into different categories for various usages. The category ‘GI illness consultations’ used in this article combined all consultations with the codes D11/diarrhoea, D70/gastrointestinal infection and D73/gastroenteritis-presumed infection.

#### *Campylobacter* data from poultry farms

The data included test results from the Norwegian surveillance programme for *Campylobacter* spp. in broiler flocks (Table 1), implemented under the responsibility of the Norwegian Food Safety Authority. There are around 500 broiler farms in Norway that are unevenly geographically distributed. Each farm may have one or more flocks at a time and each flock is usually slaughtered between 31 to 48 days old [24]. All broiler flocks younger than 50 days of age slaughtered annually between 1 May and 31 October were sampled. Sampling did not occur from November to April because of a negligible proportion of *Campylobacter* detected in winter months. For each flock, one sample consisting of 10 pooled swabs from fresh faecal/caecal droppings were collected by the farmer; the farmer was responsible for submitting the sample and its metadata. The Norwegian Veterinary Institute (NVI) performed the analysis for *Campylobacter* spp in the samples by real-time PCR [25]. Surveillance in 2020 showed that a total of 115 flocks (6.1%) tested positive for Campylobacter spp [24].

The data were cleaned for registrations where the municipality of the broiler flock was unknown (n = 11) and samples originating from flocks where the species and/or production type were not specified (n = 127). Altogether 26,435 samples from week 30 2010 to week 11 2022 were included in the data. For each ISO year, ISO week and municipality, the total number of chicken farms and the number of samples categorised into the *Campylobacter* test results; data for positive, negative, rejected, and received (i.e. not analysed yet) samples were aggregated and reported.

#### Weather data

Weather data were continuously collected from approximately 320 different measurement stations throughout Norway by the Norwegian Meteorological Institute [26], where they were analysed with an interpolative method to give daily precipitation, which includes rainfall and snowfall (mm), and daily minimum, maximum and average temperature (°C) for 1 km^2^ grid data [27]. The data were retrieved automatically on a daily basis by NIPH where they are converted to municipality averages.

### Spatio-temporal modelling

**Figure 1:**
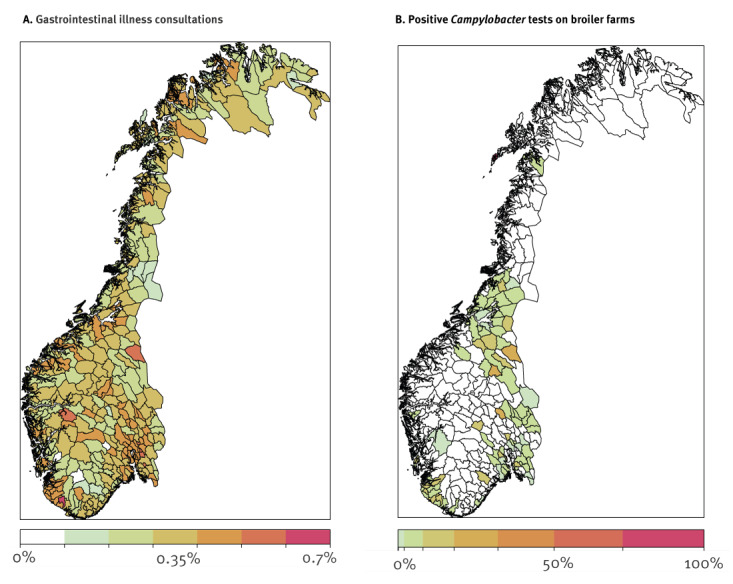
Average percentage of A. gastrointestinal illness consultations of all consultations within each municipality and B. positive *Campylobacter* tests on farms aggregated by municipality, Norway, week 30 2010–week 46 2021 (n = 356 municipalities)

We fit spatio-temporal models to weekly counts of GI consultations within all 356 Norwegian municipalities from week 30 2010 to week 11 2022, the most current week available at the time of writing, for a total of 215,024 municipality-week observations [28-31]. Model fits were determined by the ‘surveillance’ package in the statistical programming language R [32,33]. We used the total number of consultations for each municipality week as an offset term to account for population size heterogeneity and variation in demand on the health system introduced by holidays and other season-associated factors operating on a short time scale. Random intercepts were used to account for municipalities’ tendencies towards higher or lower proportions of GI consultations (Figure 1A).

We modelled these data as a negative binomial distributed outcome with a linear time trend, cyclic seasonal terms on 6 degrees of freedom (df), and separate intercept and time trends to account for the post-COVID-19 pandemic onset time window. We explored the use of the proportion of the week that was holiday and autoregressive terms, but found these were either not statistically significant after introduction of the offset term or untenable from the perspective of nowcasting because of lag time in health system data availability. The model ultimately was a special case of that described in [28,31,34] with:


$$\log\mu_{it}=\log\eta_{it}+\log\nu_{it}$$

for negative binomial distributed mean µ_it_ of observed outcome y_it _(GI illness consultations) for municipality *i* at week *t*, and offset ν_it_ for total consultations, where endemic component η_it_ is modelled with


$$\eta_{it}=\;\alpha_{i}+\alpha_{C}\cdot I(t>15.3.2020)+\beta t+\beta_{C}t\cdot I(t>15.3.2020)+\;\Sigma_{s\in S}\gamma_{s}\sin\omega_{s}t+\delta_{s}\cos\omega_{s}t$$

for municipality-specific random intercepts α_i_, time trend β, ‘COVID-19 era’ time trend β_C_ and intercept α_C_, and seasonality parameters δ_S_ and γ_S,_ with ω_S_ = (2πs)/52 for index set S, in our case [1…3]. The COVID-19 era time trend and intercept applied to the period after 15 March 2020 and was intended to account for the COVID-19 pandemic’s significant influence on the use of health system resources. Likewise, modelling the logged mean of GI consultations with log µ_it_ = log η_it_ + log ν_it_ has the benefit of allowing one to interpret parameters in terms of their expected influence on the proportion of GI illness consultations, where variables are associated with changes on the multiplicative scale of this proportion. This quantity is more stable over season, holiday period, and the time window under consideration, and one also benefits from more statistically efficient estimation of model parameters when using this parametrisation due to offset ν_it_.

The model has an extra df to account for overdispersion in the outcome. The model parametrisation has 

${Var\left(y_{it}\right)=u}_{it}+\psi \cdot \mu_{it}^{2}$

for parameter Ψ estimated from the data. We also weighted the municipality-weeks in the model according to the total number of consultations for that week in thousands, where a minimum value was set at 1. In this way, model fits reflected municipality size while incorporating the statistical associations present in smaller municipalities. We performed chi-squared tests on the season components jointly on 6 df and also 1 df tests on time trends, the COVID-19 era intercept term, and overdispersion parameter, and evaluated model calibration via use of the probability integral transformation (PIT) on ‘one-step ahead’ out-of-sample observations over the most recent 6 months of data available [35]. This assessment is performed under a scheme whereby future observations are transformed using a PIT calculated only on historical data, which should be distributed uniform under a well-calibrated model and moves progressively forward in time as one makes the assessment over a specific time interval. Adjusted McFadden pseudo R^2^ for the baseline model was estimated.

After fitting this baseline model using maximum likelihood in the R statistical environment, we built a logistic regression model for the deviation of the model’s standardised residuals falling above a specified threshold, a binary indicator of a possible GI illness outbreak of at least modest severity. It also accommodated the hypothesis that the ‘GI illness consultations’ outcome is a mixture distribution of normal GI illness dynamics and a small number of ‘outbreak’ events when possible *Campylobacter* contamination occurs, which manifests in the tail of the residual distribution. We defined peaks by those occurring above the 99^th^ percentile of that expected for the municipality-week according to the baseline model to address the trade-off between modelling true signal of a GI illness outbreak and balance and statistical power of the outcome.

For 102 municipalities with at least 10 *Campylobacter* surveillance observations and a non-zero number of encounters recorded in the health system over 11 years of data, we modelled this binary outcome using a logistic regression model with weights as used in the negative binomial model and explored distributed lag models of 1- to 5-week lags, depending on the variable, of positive tests for *Campylobacter* (Figure 1B) and municipality farm data in addition to local weather features. Nowcasting further in the future was not extensively explored partly because larger lags were not significant in models. Additionally, the hypothesised route to contamination of the general population would be expected to only be detectable statistically on a shorter timeline as longer ones would exhibit greater variability in time to outbreak which could not be isolated to a specific week lag in a model. We fit the model with robust standard errors to account for any residual correlation from the baseline model between proximal municipalities. The total number of *Campylobacter* municipality-weeks modelled was 10,667 over the 11 years of follow-up time. 

*Campylobacter* covariates explored included the proportion and total number of farms within a municipality testing positive for *Campylobacter,* and the total number of farms and number of *Campylobacter* samples taken in a municipality. The weather features we explored in models were average temperature, average precipitation, within-week temperature variation and range, and an indicator for below freezing temperatures, a subset of which have been shown to influence *Campylobacter* in broiler flocks [5,11,16,17,36]. For municipalities without *Campylobacter* surveillance data because of a lack of broiler farms, we modelled the outcome with the same weather features. Doing so assumes that all *Campylobacter* measurements are some population average, invariant across municipality and therefore absorbed into the intercept term of the model. Model selection was performed with Akaike’s information criterion (AIC), which asymptotically minimises out of sample mean square error, and chi-squared tests were performed on all included features [37].

### Sykdomspulsen One Health website

Sykdomspulsen is an umbrella term for a surveillance system and an interactive website which disseminates the data and model-based results of that system (https://docs.sykdomspulsen.no), both of which were developed at NIPH and are built on R [33]. The Sykdomspulsen surveillance system performs real-time analysis and disease surveillance of several different infectious diseases and causes of death.

While Sykdomspulsen has broader application, for this particular One Health project the system automatically includes new data (GI consultation data for human illness, *Campylobacter* data from broiler farms and weather data), runs models, and adds results to the One Health Sykdomspulsen website once a day. The One Health Sykdomspulsen website is a closed website for stakeholders in Norway. It is developed using the R shiny package, which allows the creation of interactive modules for data visualisation [33,38]. Data and model results are shown in two different ways: maps and graphs. The maps are interactive, allowing the user to zoom in and out of a desired area and hover the mouse over a municipality to reveal specific information about it. The graphs are also interactive, allowing the user to select the geographic area of interest and the time period to display. In addition, we changed the default layout and visual design through a custom cascading style sheet (CSS) to make the website more user-friendly and coherent with the visual style of NIPH.

## Results

### Parameter estimates and hypothesis testing

We modelled counts of GI consultations within all 356 Norwegian municipalities from week 30 2010 to week 11 2022, for a total of 215,024 municipality-week observations. The baseline model for the number of GI consultations (Table 2) reveals all model parameters, shown on log scale, to be highly significant. The adjusted McFadden pseudo R^2^ for the model was 0.28 [39]. The time trend prior to the start of the COVID-19 pandemic in Norway is estimated as slightly negative, indicating a gradual movement towards a slightly lower proportion of GI illnesses over time, while the COVID-19 era intercept term is also negative with a large effect size indicating an approximate 46% drop in the GI consultation proportion in the immediate period after the COVID-19 pandemic began. Visual inspection of the composition of season terms indicates expected tendencies of a lower proportion of GI consultations in spring and fall. A joint test of these terms was performed using a chi-squared null distribution on 6 df and was found highly significant (p < 1e−14). The variance of random effects is large, indicating a nearly threefold difference in the expected GI proportion between the 2.5% and 97.5% municipality quantiles, which is consistent with empirical variation in GI consultation proportion by municipality. The model ‘one-step ahead’ assessment of model calibration revealed a well-fitting model, with the cumulative distribution function’s transformation of out-of-sample observations of the 6 most recent months of data yielding an approximate uniform distribution as desired, with an only modest increase in mass above expected near 1 (Supplement S1: Probability Integral Transform figure for assessment of model calibration). We found that the PIT looked nearly identically uniform with municipality-specific variance parameters, but prioritised model parsimony to this overly flexible model.

**Table 2:** Model parameter estimates for season, time and intercept terms composing the model for gastrointestinal illness consultations over all municipalities, Norway, week 30 2010–week 11 2022 (n = 356 municipalities)

Covariate	Parameter estimate	95% CI	p value
Intercept	−5.11	−5.120 to −5.100	< 0.0001
Time trend	−0.017	−0.019 to −0.015	< 0.0001
Seasonal components	NA	NA	< 0.0001
Sine (2*pi*time/52)	0.047	0.043 to 0.052	NA
Cosine (2*pi*time/52)	0.017	0.013 to 0.022	
Sine (4*pi*time/52)	0.036	0.031 to 0.040	
Cosine (4*pi*time/52)	0.120	0.110 to 0.120	
Sine (6*pi*time/52)	0.004	−0.001 to 0.007	
Cosine (6*pi*time/52)	0.061	0.057 to 0.066	
COVID-19 era intercept	−0.63	−0.650 to −0.610	< 0.0001
COVID-19 era time trend	0.063	0.030 to 0.096	< 0.0001
Random effect variance	0.058	NA	NA
Overdispersion parameter	0.070	0.068 to 0.073	< 0.0001

CI: confidence interval; COVID-19: coronavirus disease; NA: not applicable; pi: 3.14.

The COVID-19 era intercept term and time trend were parametrised to begin 15 March 2020.

### Risk model for deviation from season-adjusted expected gastrointestinal illness consultations

The second-stage logistic regression model for the dichotomised positive deviations from the baseline model among municipalities with broiler flocks examined for *Campylobacter* revealed additional, statistically significant associations (Table 3), where odds ratios, confidence intervals, risk ratios and p values are presented. Lagged covariates from 1 to 3 weeks were all present after model selection, with the 2-week-lagged proportion of positive *Campylobacter* tests and number of broiler farms in a municipality both associated with an expected increase in the probability of an outbreak. The number of *Campylobacter* samples taken is also significant and has an odds ratio (OR) of less than 1. Because of the presence of the positive *Campylobacter* proportion in the same model, increasing the number of *Campylobacter* samples, holding all other variables constant, indicates a lower positive *Campylobacter* proportion; thus, a smaller risk of the outcome is expected. Because the outbreak probability is low, exponentiated coefficients can be interpreted on the risk ratio scale, with that of *Campylobacter* presence vs absence to be 1.73. Precipitation, temperature and their transformations were likewise associated with changes in the outbreak probability. Risk ratios are presented for these continuous covariates over their 2.5^th^ and 97.5^th^ quantiles, which give the change in risk over the range of realised covariate values being analysed. For example, the interpretation of precipitation (mm) at its 2-week lag is, for each additional 1 mm, one expects 3% higher risk of an outbreak as defined by extreme deviation from the baseline model (p = 0.013). Additionally, at the 97.5% quantile of that covariate, the risk of an outbreak is 1.49 times higher than at the 2.5% quantile. Straightforward interpretation of other weather covariates is difficult because of the association between, for example, the presence of freezing temperatures and the variation of temperature within a week. The model explained a statistically significant amount of variation in the outbreak outcome, with fitted probabilities of an outbreak varying from 1.4% to 4.03% in the 2.5^th^ to 97.5^th^ quantiles.

**Table 3:** Results from risk model for gastrointestinal illness consultations’ deviation from baseline model over municipalities with *Campylobacter* data, Norway, week 30 2010–week 11 2022 (n = 102 municipalities)

Covariate (lag)	Lag	OR	95% CI	Risk ratio over covariate quantiles	p value
Intercept	NA	0.015	0.012 to 0.020	NA	< 0.001
Temperature SD	1 week	1.136	1.03 to 1.26	1.91	0.014
Freezing temperature	1 week	1.748	1.09 to 2.80	1.54	0.021
Number of *Campylobacter* samples taken	1 week	0.938	0.882 to 0.99	0.62	0.040
Precipitation (mm)	2 weeks	1.027	1.005 to 1.05	1.49	0.013
Freezing temperature	3 weeks	0.359	0.085 to 1.51	0.66	0.163
*Campylobacter* proportion	2 weeks	1.004	1.0002 to 1.009	1.73	0.036
Number of municipality flocks	2 weeks	1.013	1.001 to 1.026	1.70	0.030

CI: confidence interval; NA: not applicable: OR: odds ratio; SD: standard deviation.

Weather and *Campylobacter* covariates were chosen to minimise Akaike’s information criterion (AIC). Weather data come from the Norwegian Meteorological Institute and *Campylobacter spp* sampling from the Norwegian surveillance programme of broiler flocks [24].

### Sykdomspulsen One Health website

**Figure 2:**
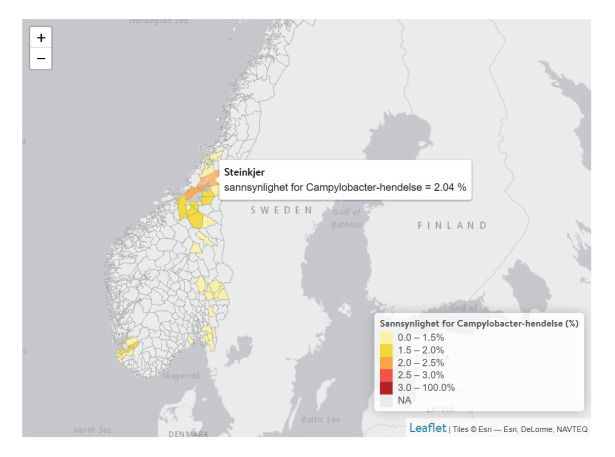
Interactive map, as presented on the Sykdomspulsen One Health website for selected municipalities, Norway, week 45 2021 (n = 38 municipalities)

**Figure 3:**
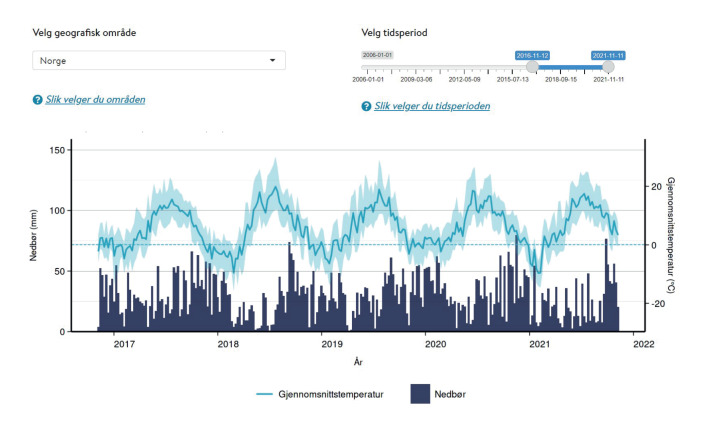
Interactive climatograph, as displayed on the Sykdomspulsen One Health website, with weekly data aggregated over all municipalities, Norway, week 45 2016 to week 45 2021 (n = 356 municipalities)

The One Health Sykdomspulsen website is used to visually present data and model output. It displays several figures including (i) a map of the number of farms per municipality, (ii) a map of the model-based risk of an outbreak happening in the current week per municipality (Figure 2), (iii) a climatograph with temperature and precipitation (Figure 3), (iv) a graph of the total number of *Campylobacter* samples and the proportion of positive samples for *Campylobacter* in poultry and (v) a graph showing both the historic proportion of physicians’ consultations related to GI illness and the nowcasted proportion for the current week. 

The map shows the probability of an outbreak event (Norwegian: Sannsynlighet for *Campylobacter*-hendelse) happening in the current week. When hovering over a municipality, the name of the municipality and the probability of an event are displayed. For sensitivity reasons, only the municipalities with three or more farms are displayed, which yields a total number of 38.

The graph shows average temperature (Norwegian: gjennomsnittstemperatur) and precipitation (Norwegian: nedbør) data. The user can choose the area (Norwegian: geografisk område) and the time period (Norwegian: tidsperiod), which will update the graph automatically.

All maps and graphs are interactive, as the user can choose the geographical area and time period to display. In addition to showing raw data and model results, the website also offers information on One Health in general, the relevant European Union (EU) projects, the partner institutions, and background information on GI illness and *Campylobacter* infections in Norway. This creates a hub of information common to the sectors of public health, animal health and food safety, where data are updated automatically, and which is easy to maintain and extend to future research programs.

This website is currently a pilot project, open to a few stakeholders (NIPH, NVI, and the Norwegian Food Safety Authorities). We are in the process of collecting feedback from these users to improve the content and usability of the website in the future.

## Discussion

In this One Health collaboration, data from the animal, human, and environmental sectors were combined in a model for nowcasting human GI illness outbreaks. Our results illustrate how data from these sectors could be used in syndromic surveillance with automated collection and combination of separate databases. Given the need for improving nowcasting of disease outbreaks, this study serves as an example for setting up a One Health system and website that can be built upon for further One Health collaboration.

Our outbreak nowcasting models yielded statistically significant relationships that are consistent with the hypothesis that weather patterns and presence of *Campylobacter* sampled on municipality broiler farms (used as a proxy for increased environmental contamination) are associated with modest increases in the GI illness consultation burden in Norwegian municipalities. In several European countries, an EFSA report claimed that broilers might account for 50–80% of human cases of campylobacteriosis. However, the consumption of contaminated broiler meat might only account for 20–30% of those cases [40]. This is also supported when analysing both broiler and human Norwegian *Campylobacte*r data where there was simultaneous space-time clustering in both broilers and humans, despite broiler-meat having a nation-wide distribution [6]. Though consumption of chicken meat is a risk factor for campylobacteriosis in Norway [41], this could indicate that broiler meat is not the only source of human infection in the country, and that there are common sources of contamination for *Campylobacter*-positive broiler flocks and human campylobacteriosis cases [6]. This is in line with the notion that while poultry products are one possible source of *Campylobacter* infections, environmental sources like drinking untreated water from streams and lakes are also significant risk factors for acquiring *Campylobacter* infection [41-45]. 

Some *Campylobacter* samples from broiler farms were excluded because of unknown municipality or missing species or production type. This is not expected to influence model results since the number of excluded samples was 138 among 26,573. Not all municipalities have broiler farms and hence many geographical regions of Norway were excluded from the model, which can influence generalisability to those regions. For those Norwegian municipalities without *Campylobacter* sampling, a model based on only weather data was developed.

The consultation data are not specifically targeted at detecting *Campylobacter* infection, only GI illness in general. There are several increases in GI consultations in the data that are not because of *Campylobacter*, but rather other infections. This reduces the statistical power of detecting genuine associations. To address this difficulty, we only used patients aged 30 to 64 years since there is an increased probability of having other GI illness like norovirus, adenovirus and sapovirus in other age groups. These viruses are very contagious when people are in close proximity, and outbreaks in nurseries, schools and care facilities for elderly people are very common. The variation in timescale on which environmental contamination would be expected to infiltrate the general population is another modelling challenge that might underpower the study because only specific lag times were searched over during model selection. The overall risk of a *Campylobacter* outbreak is low, indicative of both generally modest deviations from seasonal expectations of the GI consultation proportion and low prevalence of such outbreaks in the country of Norway. However, the model still provides a useful tool and proof of concept for the promise of combining and modelling these disparate data sources and communicating with policymakers in turn. It also points to improvement of nowcasting *Campylobacter* outbreaks in humans with the addition of other data sources or specification of more defined population groups.

Our model fits are consistent with current understanding of *Campylobacter*, weather, and GI illness, but also give insight into their interplay since these factors have mainly been studied pairwise in the past. The model shows that greater numbers of broiler flocks testing positive for *Campylobacter* at a 2-week lag is associated with modest increases in risk for an outbreak in humans. It is notable that *Campylobacter* presence at other time lags was not statistically significant in the model. However, the study was not designed to identify any causal relationship between *Campylobacter* infection in poultry and humans and one should be careful interpreting the statistical associations identified as such. While it is known that weather influences likelihood of testing positive for *Campylobacter*, the influence of weather on GI illness after controlling for a given amount of *Campylobacter* has not been studied, which is the interpretation of weather covariates in this multivariate regression model. Precipitation and temperature at different time lags are likewise statistically significant in the model, with effect sizes indicating their increased and reduced association with an outbreak depending on the lag and variable. Interpretation of these covariates is difficult since each is conditional on the other, and there is temporal correlation between lagged values. It is clear, however, that weather events have significant bearing on the outcome.

The model points to several ways in which the surveillance system could be improved to better understand the interaction of *Campylobacter*, environment, weather and human health, and to communicate those insights to health professionals. There is clear nowcasting benefit to the availability of more *Campylobacter* testing results. This study uses already available information from *Campylobacter* surveillance in broiler flocks, the sampling of which is meant to identify and restrict the entry of *Campylobacter* into the food chain. Because broiler farms are therefore used as a proxy for environmental *Campylobacter* contamination, it could also be beneficial to survey environmental contamination directly, including data from the control of water systems and incorporate it into an expanded model.

The One Health Sykdomspulsen website is based on a collaboration between public health, animal health, and the food safety authority and is therefore an important step into a common One Health surveillance programme of infectious disease. The website is in a pilot stage, and its use by health authorities (NIPH, NVI, and the Norwegian food safety authority) will continue to evaluate and develop. We therefore anticipate that such a website, accessible to various sectors involved in infection prevention, with automated, near real-time surveillance of *Campylobacter* and synchronous with other infectious diseases, will prove to be useful in the daily surveillance, understanding, and prevention of zoonotic illnesses. At a later stage, for example, municipal doctors may also be introduced to the website and be notified when there is an increased risk of *Campylobacter* outbreak. Through this channel, health authorities could subsequently advise the local water management to test water quality, increase disinfection of the water and/or notify the public to boil their water.

## Conclusion

This study demonstrates the potential benefits of integrating data from animal and human sectors via statistical models when setting up a One Health system and website to improve disease surveillance. We also show how automated, regular, and coordinated data transfer between animal and public health institutions can facilitate the kind of model building required for achieving this goal. The communication of model insights to health professionals via a website is the last link in a surveillance system with practical use to society at large. This project is therefore a step towards improving the One Health collaboration in Norway and solidifying ways of working across institutions. As part of a wider EU project, it is also an important development for One Health surveillance in Europe.

## Supplementary Data

78000 Supplement
