# Supplementary material for: A One Health real-time surveillance system for nowcasting Campylobacter gastrointestinal illness outbreaks, Norway, week 30 2010 to week 11 2022
Source: Euro Surveill. 2022 Oct 27;27(43):2101121. doi: 10.2807/1560-7917.ES.2022.27.43.2101121 (PMC9615412; doi:10.2807/1560-7917.ES.2022.27.43.2101121)
Supplement: Supplement [file 21-01121_SWANSON_SUPPLEMENT.pdf]

S1: Probability Integral Transform figure for assessment of model calibration

This supplementary material is hosted by *Eurosurveillance* as supporting information alongside the article "A One Health real-time surveillance system for nowcasting *Campylobacter* gastrointestinal outbreaks, Norway, week 30 2010 to week 11 2022" on behalf of the authors who remain responsible for the accuracy and appropriateness of the content. The same standards for ethics, copyright, attributions and permissions as for the article apply. Supplements are not edited by Eurosurveillance and the journal is not responsible for the maintenance of any links or email addresses provided therein.

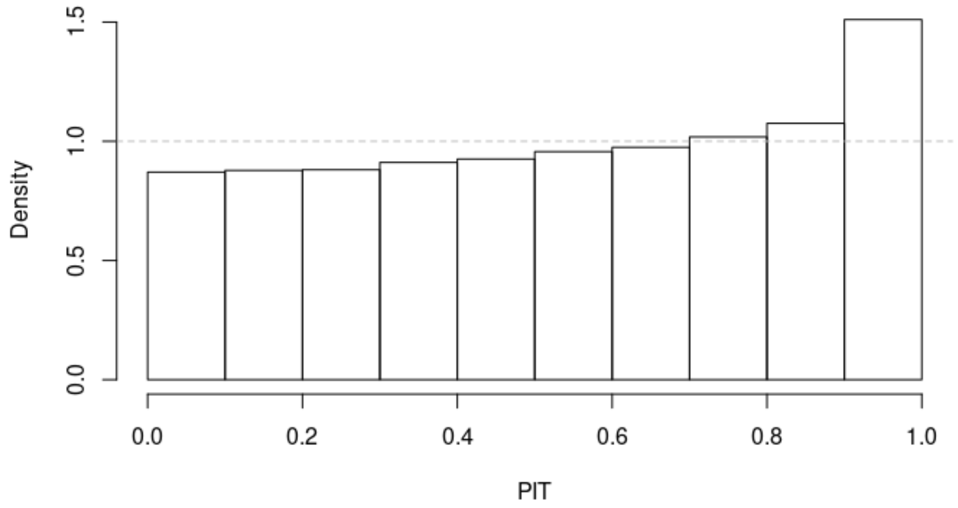

Figure 1: PIT plot for model calibration
